# Multifunctional Janus Nanoparticles Capable of Anchoring to the Cell Membrane and Serving as “Cellular Backpacks” for Advanced Theranostics

**DOI:** 10.1021/jacs.5c02587

**Published:** 2025-04-05

**Authors:** Min Hao, Yidan Chen, Johannes Leisen, Ted J. Whitworth, Younan Xia

**Affiliations:** † The Wallace H. Coulter Department of Biomedical Engineering, Georgia Institute of Technology and Emory University, Atlanta, Georgia 30332, United States; ‡ School of Materials Science and Engineering, Georgia Institute of Technology, Atlanta, Georgia 30332, United States; § School of Chemistry and Biochemistry, Georgia Institute of Technology, Atlanta, Georgia 30332, United States; ∥ Robert P. Apkarian Integrated Electron Microscopy Core, Emory University, Atlanta, Georgia 30322, United States

## Abstract

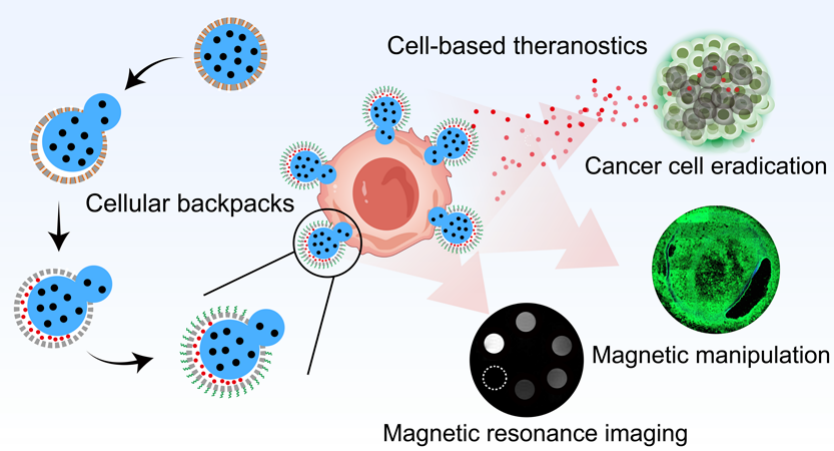

A cell-based theranostic system can be fabricated by attaching nanomedicines to the surface of carrier cells, but it remains a challenge to achieve the attachment without involving endocytosis. Herein, we address this challenge by developing multifunctional Janus nanoparticles with orthogonal surface properties for the two opposite halves. When incubated with carrier cells, the hydrophobic half made of polystyrene readily inserts into the plasma membrane, whereas the hydrophilic SiO_2_ half grafted with poly(ethylene glycol) protrudes away from the cell surface. Additionally, the SiO_2_ half can be made with a cavity to hold theranostic agents and thus serves as a “backpack” for the carrier cell. By confining the theranostic agents in the SiO_2_ compartment and outside the carrier cell during the delivery process, their adverse impact on the cell is minimized. Upon release in an in vitro spheroid model, the agents quickly eradicate cancer cells. Moreover, the polystyrene half can be loaded with superparamagnetic nanoparticles to enhance magnetic resonance imaging contrast and enable magnetic manipulation, facilitating image-guided and target-directed treatments. By further optimizing the interactions between the multifunctional Janus nanoparticles and carrier cells, this system can be developed into a robust platform for cell-based theranostics.

## Introduction

The remarkable capability of cells to sense and respond to environmental stimuli has made cell-based therapies a transformative approach for addressing previously intractable diseases such as hematologic malignancies and neurodegenerative disorders.^1,2^ Among the cell-based therapies, the “Trojan horse” approach is particularly intriguing and promising as it can leverage the innate properties of carrier cells to efficiently transport therapeutic agents across biological barriers, enabling precise and localized delivery to the site of interest.^3,4^ The therapeutic agents, including small molecule drugs, nucleic acids, peptides, proteins, and various types of nanomedicines, are typically preloaded into the carrier cells through endocytosis.^5^ In this case, it has been a grand challenge to manipulate the complex pharmacokinetics of the therapeutic agents and thereby minimize their adverse impacts on the carrier cells.^6^ Alternatively, the therapeutic agents can be preloaded into nanoparticles to be attached to the plasma membrane, eliminating any possible harm to the carrier cells.^7,8^ To this end, it is of key importance to optimize the interaction between the nanoparticles and the cell membrane to ensure that the nanoparticles only attach to the outer surface without invoking endocytosis for the fabrication of “cellular backpacks”.

Owing to their immune-regulating ability and multidirectional differentiation potential, mesenchymal stem cells (MSCs) have emerged as a versatile platform for developing cell-based therapies.^9−11^ Through physical adsorption or covalent conjugation, it is possible to engineer the surface properties of nanoparticles and thus control their spatial distributions relative to the plasma membrane.^12−14^ In one study, Cheng and co-workers developed NeutrAvidin-coated nanoparticles capable of attaching to MSCs possessing biotinylated plasma membrane.^15^ Similarly, Liang and co-workers reported an antigen–antibody bioconjugation strategy to help anchor nanoparticles to the surface of MSCs.^16^ While these methods can effectively attach nanoparticles, their reliance on ligand–receptor binding for cell membrane attachment limits their applicability and may lead to receptor-mediated endocytosis.^17,18^ It would be a major step forward if we have nanoparticles capable of directly anchoring to the cell membrane without involving ligand–receptor interactions. To this end, Janus nanoparticles hold promise as a strong candidate because their two opposite halves can be readily prepared with orthogonal surface properties.^19,20^ In principle, the two halves can be made favorable for and resistant to endocytosis, respectively, allowing the Janus nanoparticle to sit right on the outer surface of a cell without activating internalization.

**Figure 1:**
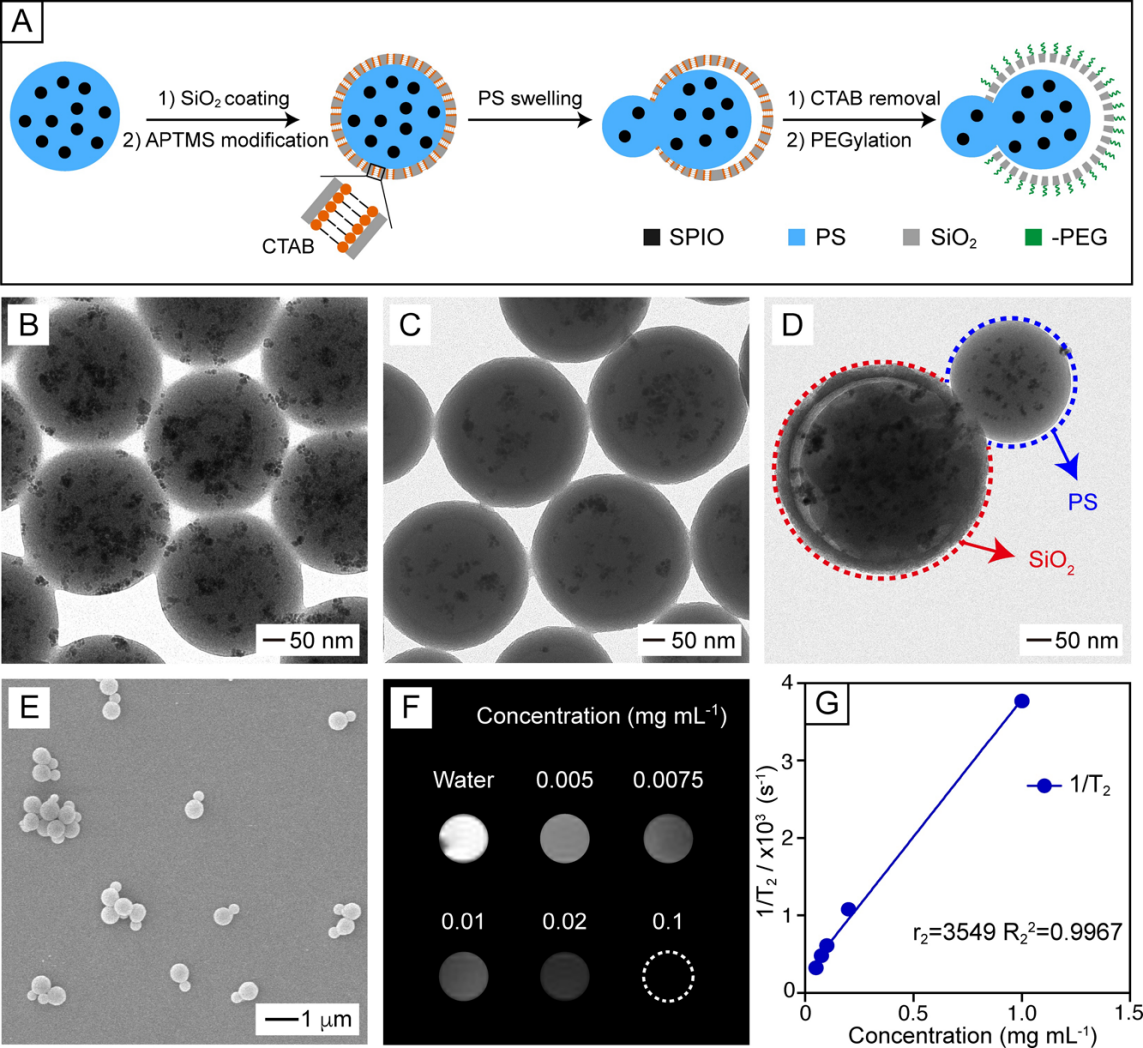
Preparation and characterizations of the multifunctional Janus nanoparticles. (A) Schematic of the synthetic protocol, including swelling of the core–shell particles with aqueous THF, followed by removal of CTAB and selective PEGylation of the SiO_2_ surface. (B–D) TEM images of (B) SPIO-PS beads, (C) (SPIO-PS)@SiO_2_ core–shell particles, and (D) multifunctional Janus nanoparticles fabricated by swelling PS with aqueous THF (30%, v/v) for 6 h, followed by quenching with ethanol, removal of CTAB, and PEGylation. (E) SEM image of the multifunctional Janus nanoparticles. (F) T_2_-weighted MRI and (G) Plot of 1/T_2_ as a function of the concentration of multifunctional Janus nanoparticles.

Here we demonstrate the rational fabrication of multifunctional Janus nanoparticles capable of anchoring to cell surface and thus serving as “cellular backpacks” for cell-based theranostics. As illustrated in Figure 1A, the nanoparticle is fabricated through swelling-induced symmetry breaking, and the resultant cavity inside the SiO_2_ half can be loaded with doxorubicin (Dox) by leveraging the mesoporous channels created in the SiO_2_ shell through surfactant-templating. The hydrophilic SiO_2_ half is then PEGylated to mitigate protein adsorption and cellular uptake, whereas the other half made of polystyrene (PS) remains hydrophobic for favorable insertion into the lipid bilayer, allowing the nanoparticle to stay right at the cell surface without triggering endocytosis. Furthermore, the incorporation of superparamagnetic iron oxide (SPIO) nanoparticles into the PS compartment enhances magnetic resonance imaging (MRI) and enables magnetic manipulation, offering a versatile platform for advanced diagnosis and treatment.

## Results and Discussion

### Synthesis and Characterizations of the Multifunctional Janus Nanoparticles

The PS beads containing SPIO nanoparticles (SPIO-PS) were synthesized using mini-emulsion polymerization.^21^ As shown by the transmission electron microscopy (TEM) images in Figures 1B and S1A, they had a uniform diameter of ca. 320 nm. The beads were then coated with a SiO_2_ shell of ca. 40 nm thick in the presence of cetyltrimethylammonium bromide (CTAB) as a porogen (Figures 1C and S1B). To ensure that PEGylation occurs exclusively on the SiO_2_ half of the Janus particles, we modified the surface of the core–shell particles with amine groups via (3-aminopropyl)trimethoxysilane (APTMS) treatment prior to swelling-induced symmetry breaking. Zeta potential analysis showed a significant increase in surface charge upon modification, confirming the presence of amine groups (Figure S2). When immersed in aqueous tetrahydrofuran (THF), the PS core would be swollen to generate an internal pressure that eventually exceeded the structural integrity of the SiO_2_ shell.^22^ As a result, an opening was punched in the shell to allow partial extrusion of the swollen PS (together with some SPIO nanoparticles), resulting in the formation of a Janus nanoparticle. With the use of 30% aqueous THF, the protruded PS half of the Janus nanoparticles was ca. 240 nm in diameter, whereas the opposing SiO_2_ half remained at ca. 400 nm in size (Figures 1D and S3). As shown by the low-magnification scanning electron microscopy (SEM) and TEM images in Figures 1E and S4, the Janus nanoparticles were reasonably uniform in size. When the concentration of THF was decreased to 20%, the diameter of the protruded PS half reduced to ca. 170 nm, demonstrating the influence of THF concentration on the swelling process (Figure S5). When the CTAB was extracted with NH_4_NO_3_ in ethanol, an array of mesoscale pores developed in the SiO_2_ shell, allowing for payload introduction in the next step.^23^ Finally, the amine groups on the SiO_2_ half were reacted with the carboxyl groups of poly(ethylene glycol) (PEG) via carbodiimide-*N*-hydroxysuccinimide (EDC-NHS) coupling, yielding the multifunctional Janus nanoparticles. Zeta potential analysis showed a decrease in surface charge (Figure S2), confirming the success of coupling.

Known for its noninvasive, nonionizing, and radiation-free characteristics, MRI has become a powerful diagnostic tool in clinics.^24^ In this regard, SPIO nanoparticles are well-established as a negative contrast agent that produces darkened signals in T_2_-weighted imaging while exhibiting a high transverse-to-longitudinal relaxivity ratio (*r*_2_/*r*_1_).^25^ To evaluate the imaging capability of the SPIO-incorporated Janus nanoparticles, we conducted a set of T_2_-weighted MRI measurements. As shown by the T_2_-weighted image in Figure 1F, the samples exhibited progressively darker signals with increasing particle concentration. As shown in Figure 1G, we quantified the *r*_2_ of the SPIO-incorporated Janus nanoparticles by plotting the inverse relaxation time (1/T_2_) as a function of particle concentration. The *r*_2_ value was determined from the slope of the plots. It is documented that the *r*_2_/*r*_1_ ratio of iron oxide-based nanoparticles is dependent on parameters such as size, shape, and surface modification.^26^ The *r*_2_/*r*_1_ ratio (ca. 44.1) of the SPIO-incorporated Janus nanoparticles (Figures 1G and S6) was much greater than those of commercial agents based on iron oxides.^27^ This result is in agreement with the finding that encapsulating SPIO in liposomes or particles composed of PEG or other polymers can improve their dispersion in an aqueous medium for an increase in contact with water and thus augmentation in T_2_ relaxivity.^26^ It is worth pointing out that the MRI performance of the Janus nanoparticles was comparable to their parental core–shell particles (Figure S7), implying that the magnetic properties of SPIO were preserved in the encapsulation and swelling processes. Next, we demonstrated their magnetic responsiveness by placing a magnet next to a vial containing the Janus nanoparticles (Figure S8). Within 1 min, the particles were rapidly attracted to one side of the vial, indicating their strong and quick response to the external magnetic field. This behavior can be attributed to the relatively high loading of SPIO in the PS component. Altogether, the Janus nanoparticles offer a sensitive handle for manipulating cellular behaviors with a magnet while harnessing the noninvasive visualization capability of MRI to enhance detection and diagnostic potential.

### Attachment of the Multifunctional Janus Nanoparticles to the Plasma Membrane

It is documented that the surface properties of a nanoparticle, including the hydrophobicity and charge (both sign and density), can significantly affect its interactions with the membrane of a cell and thus control its fate in terms of endocytosis.^28^ We first evaluated the biocompatibility of the PEGylated particles with MSCs and no adverse impacts was detected (Figure S9). To understand whether the orthogonal surface properties of the multifunctional Janus nanoparticles allow them to attach to the cell membrane without being internalized, we labeled the particles using rhodamine B by loading them into the cavity of the SiO_2_ compartment prior to surface PEGylation. The particles without and with PEGylation were denoted as JNPs and P-JNPs, respectively. The merged bright-field and fluorescence micrographs showed the colocalization of red fluorescence from rhodamine B with the particles, confirming their successful labeling (Figure S10). We then incubated the particles with MSCs, fibroblasts, and Hela cells, respectively, for different periods of time and analyzed their spatial distributions relative to the plasma membrane using confocal laser scanning microscopy.

**Figure 2:**
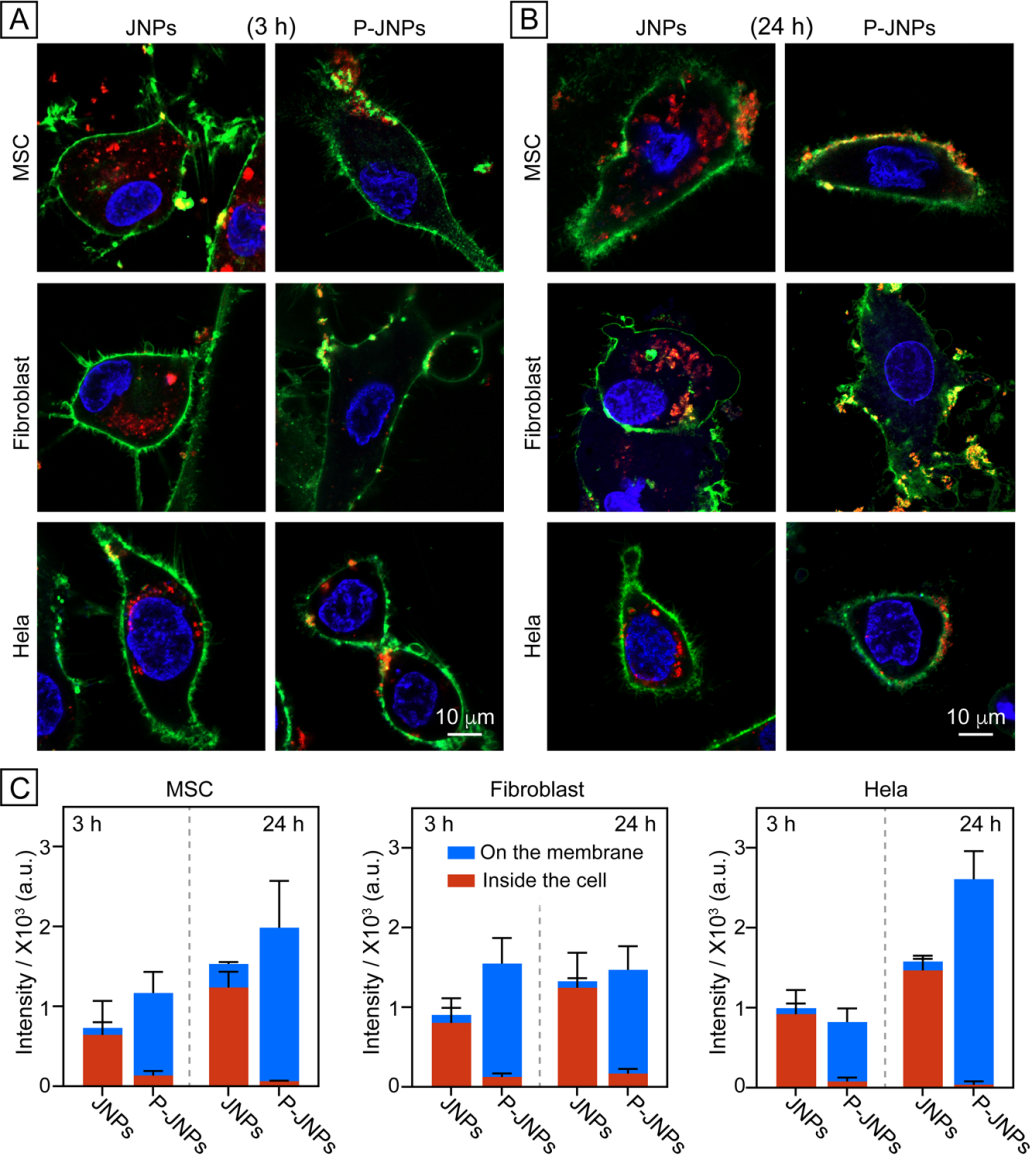
Spatial distributions of the Janus nanoparticles relative to the cell membrane. (A,B) Confocal fluorescence micrographs of the cells incubated with the two types of Janus nanoparticles (red) for (A) 3 and (B) 24 h, respectively, followed by staining of the plasma membrane with CellBritesteady (green) and nucleus with Hoechst 33342 (blue). (C) A quantitative analysis of the distributions of the Janus nanoparticles situated on the plasma membrane and inside the cell, respectively. Data are shown as mean ± standard deviation (SD) (*n* = 10).

As shown by the confocal micrographs in Figure 2A, there was only minimal overlap between the red fluorescence from the rhodamine B in the JNPs and the green fluorescence from the cell membrane after incubation with MSCs for 3 h. With PEGylation, in contrast, the red fluorescence showed significant overlap with the green fluorescence at *t* = 3 h, suggesting that the P-JNPs preferentially anchored to the cell membrane. When the incubation time was prolonged to 24 h (Figure 2B), the JNPs were mainly located inside the cells, suggesting that the majority of JNPs had been internalized by the cells. More importantly, P-JNPs remained membrane-associated, exhibiting minimal internalization and sustained surface retention. A quantitative analysis of the micrographs (Figure 2C) indicated that the fluorescence intensities of the P-JNPs on the MSC membrane were ca. 12.1- and 6.5-fold greater at *t* = 3 and 24 h, respectively, relative to those of the JNPs. Conversely, the intracellular fluorescence intensities of the P-JNPs were markedly lower, approximately 18 and 5% of the corresponding values of the JNPs. A similar trend was also observed in other types of cells tested, including fibroblasts and Hela cells, demonstrating that the orthogonal surface properties indeed made the multifunctional Janus nanoparticles preferentially anchor to and stay at the outer surface of the plasma membrane regardless of cell phenotype. Interestingly, the intracellular retention of the JNPs showed a progressive increase across the three cell types tested, with no discernible extracellular accumulation observed over time. In contrast, the P-JNPs showed persistent membrane association and negligible internalization. These findings suggested that exocytosis was minimal or absent for both the JNPs and P-JNPs.

When the diameter of the protruded PS half of the P-JNPs was reduced from 240 to 170 nm, we still observed a significant level of colocalization with the plasma membrane across all the three cell types tested after 24 h of incubation (Figure S11). However, the particles with a smaller protruded PS half showed a lower membrane-associated fluorescence intensity. When compared with the data shown in Figure 2, it is clear that a larger hydrophobic PS half would make P-JNPs more favorable for anchoring to the plasma membrane. Moreover, a larger PS protrusion is advantageous for hosting a larger amount of payload because it corresponds to a larger cavity in the SiO_2_ shell. As such, we decided to focus on the Janus particles with a larger PS half (ca. 240 nm) for all the remaining in vitro tests.

We further evaluated the spatial distributions of the rhodamine B-labeled Janus nanoparticles relative to the membrane of macrophage by incubating them with RAW 264.7 cells for 3 and 24 h (Figure S12). Again, the JNPs were primarily localized inside the cells, whereas the P-JNPs remained on the plasma membrane at both time points (Figure S12A). The profiles of fluorescence intensities in Figure S12B demonstrated negligible colocalization between JNPs and the plasma membrane, whereas P-JNPs exhibited an extensive overlap. These findings further confirmed the sustained association of P-JNPs with the plasma membrane to markedly reduce their chance of being internalized by the cells.

**Figure 3:**
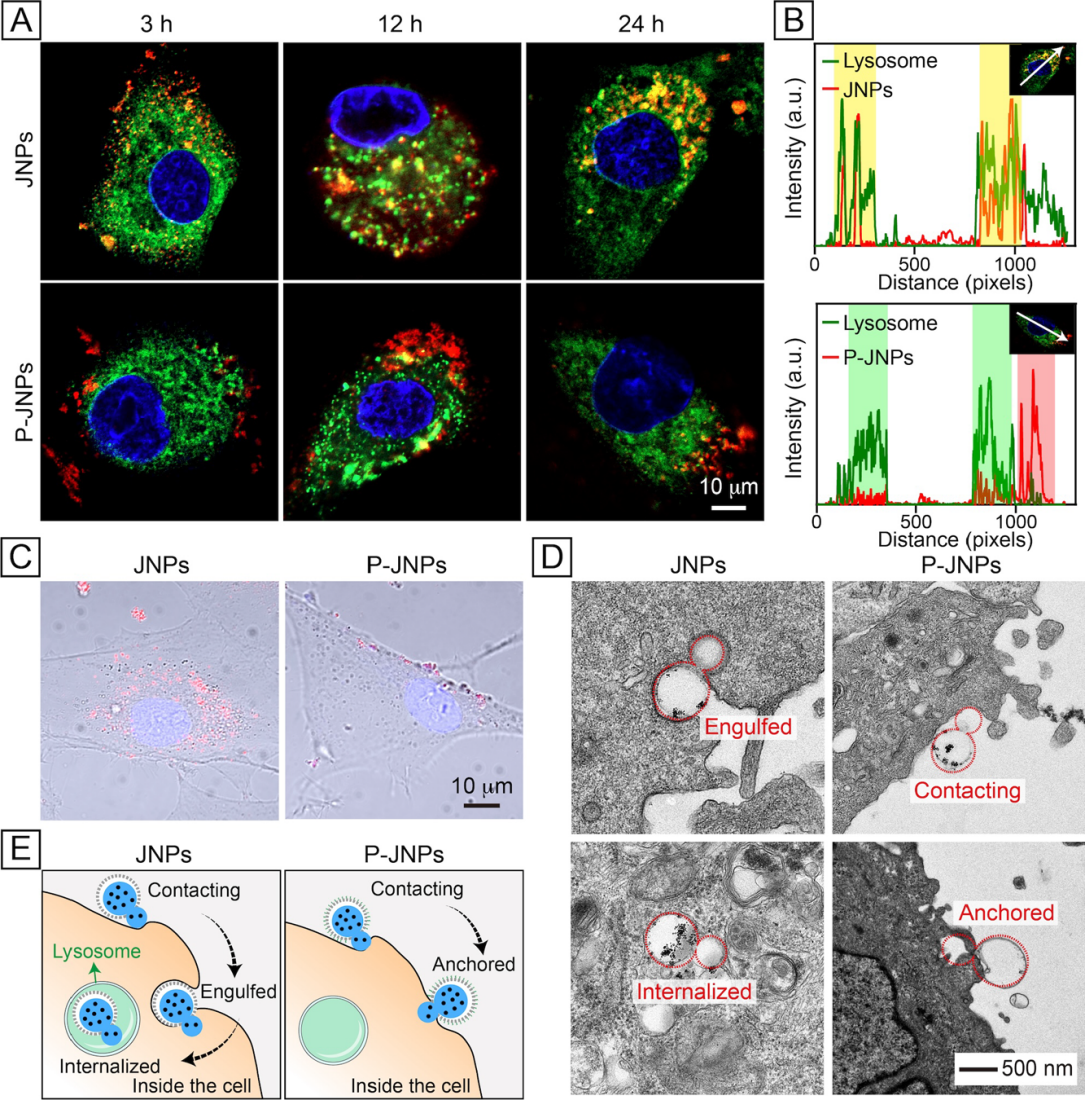
Spatial correlation between the particles and lysosomes. (A) Confocal micrographs of the MSCs after incubation with the two types of particles (red) for 3, 12, and 24 h, respectively, followed by staining of lysosomes and nucleus with Lyso-tracker (green) and Hoechst 33342 (blue), respectively. (B) Profiles of fluorescence intensities of the two types of particles, as well as the lysosomes in an individual cell, after 24 h of incubation. The fluorescence profiles correspond to the white arrows in the insets. The red, green, and yellow-colored boxes represent the primary fluorescence distribution regions of the particles, lysosomes, and their overlap, respectively. (C) Merged bright-field and fluorescence micrographs of the MSCs after incubation with the two types of particles (red) for 24 h. (D) TEM images of the MSCs after incubation with the two types of particles (outlined by red contours) for 3 h. (E) Schematic comparing the interactions of the two types of particles with a cell.

To further investigate the impact of the orthogonal surface properties on the internalization behavior of the Janus nanoparticles, we incubated the rhodamine B-labeled particles with MSCs for different periods of time and then analyzed the spatial distributions of these particles relative to the lysosome (Figure 3A). Upon internalization, the nanoparticles are typically transported to early endosomes and subsequently to lysosomes.^29,30^ Over time, the red fluorescence from the JNPs progressively overlapped with the green fluorescence from lysosomes, whereas the P-JNPs showed minimal colocalization. These results indicate that the JNPs were internalized by the cells and then transported to lysosomes, whereas the P-JNPs could remain stably anchored to the plasma membrane to show effective reduction in cellular internalization. Notably, neither JNPs nor P-JNPs exhibited endosomal escape. The profiles of fluorescence intensities in Figure 3B confirmed that the orthogonal surface properties augmented by PEGylation significantly reduced cellular internalization. A direct comparison of the merged bright-field and fluorescence micrographs demonstrated that PEGylation effectively shifted the location of the particle from intracellular to predominantly membrane-associated (Figure 3C).

We also used TEM to analyze the interactions between the Janus particles and cells (Figures 3D and S13). Again, the JNPs were readily engulfed and internalized by the cell, and only the PS component of the P-JNPs was inserted into the plasma membrane while leaving the PEGylated compartment to point away from the cell surface. Figure 3E shows a comparison of the distinct behaviors of the JNP and P-JNP when they interact with cells. According to the results from a computational investigation of how hydrophobic nanoparticles interact with the lipid bilayer,^31^ we further show a schematic in Figure S14 to illustrate how the hydrophobic PS half of the P-JNP is inserted into and anchored to the lipid bilayer. Upon contacting, the hydrophobic PS half induces disorder in the lipid headgroups, followed by forming hydrophobic interaction with the lipid tails.^31^ These interactions collectively drive the preferential insertion of the PS half into the lipid bilayer. In contrast, for the hydrophilic SiO_2_ half, the PEG possesses long and flexible chains that form a highly hydrated, dynamic, brush-like coating on the surface.^32^ It is documented in the literature that PEGylation can provide a steric hindrance to keep the surface away from the cell membrane.^28^ Additionally, the heavily hydrated backbone and the inert methoxy-terminal group of the PEG we used also contribute to the suppression of nonspecific protein adsorption.^33^ As a result, the hydrophilic SiO_2_ half covered by PEG can prevent cellular internalization.

### Mitigation of the Adverse Impacts of Therapeutic Agents on Carrier Cells

**Figure 4:**
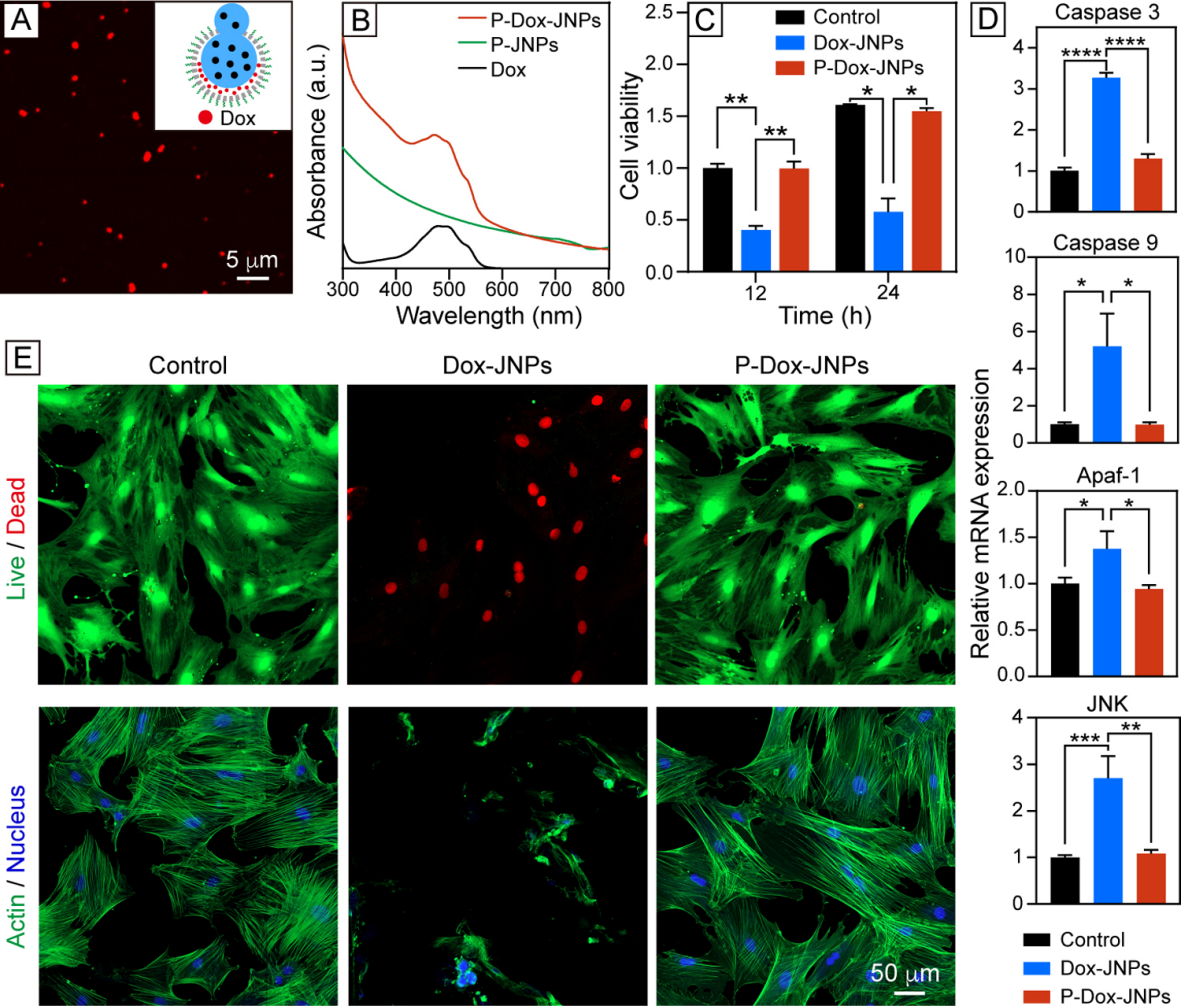
Characterizations of the Dox-loaded Janus particles and their Dox-induced toxicity toward cells. (A) Schematic (inset) and fluorescence micrograph of the P-Dox-JNPs. (B) UV–vis spectra of the PEGylated particles without and with the loading of Dox. (C) Cell counting kit 8 assay evaluating MSC viability after incubation with the two types of Dox-loaded particles for 12 and 24 h, respectively. Control: untreated MSCs. Data are normalized to the control group at 12 h and are presented as mean ± SD (*n* = 3). Two-way analysis of variance (ANOVA) with Tukey’s multiple comparisons test (*p < 0.05 and **p < 0.01). (D) RT-qPCR analysis of apoptosis-specific markers in the MSCs after incubation with the two types of Dox-loaded particles for 24 h (*n* = 3). One-way ANOVA with Tukey’s multiple comparisons test (*p < 0.05, **p < 0.01, ***p < 0.001, and ****p < 0.0001). (E) Confocal micrographs of the MSCs incubated with the two types of Dox-loaded particles for 24 h, followed by live/dead staining or actin/nucleus staining.

Conventional nanoparticles, such as those based on lipids and polymeric or inorganic materials, are widely used as carriers for drug delivery due to their high biocompatibility, tunable size, and the ability to protect the drug.^34^ Typically, these nanoparticles are loaded into the carrier cells or camouflaged with cell membranes to enable efficient drug delivery.^7^ However, the nanoparticles inside the carrier cells are inevitably exposed to intracellular enzymes, leading to premature release of the drug and thus exerting unwanted cytotoxicity on the carrier cells. It is also an intense and complicated job to extract and then coat the cell membrane, not mentioning the challenges in standardizing the composition and functionality. Here, the P-JNPs, powered by rational fabrication and the membrane-anchoring capability, allow us to mitigate the drug-induced adverse impact on carrier cells. To evaluate the effectiveness, a model drug such as Dox^35^ was loaded into the cavity of the SiO_2_ compartment prior to surface PEGylation. The resultant particles showed red fluorescence and a characteristic ultraviolet–visible (UV–vis) absorption peak similar to that of free Dox (Figure 4A,B), confirming the successful loading of Dox. We then compared the cytotoxicity of the Dox-loaded particles, without and with PEGylation (donated Dox-JNPs and P-Dox-JNPs), by analyzing the viability of the cells (Figures 4C and S15A). The cells incubated with the Dox-JNPs showed the lowest viability across the three samples, indicating their inability to mitigate the deleterious effects arising from the loaded Dox. Upon surface PEGylation, the adverse impacts became negligible.

Next, we performed reverse transcription-quantitative polymerase chain reaction (RT-qPCR) to evaluate the expression of apoptosis-related markers in the cells (Figure 4D). Caspase 3 and Caspase 9 are central mediators of apoptosis, while apoptotic protease activating factor-1 (Apaf-1) and c-Jun N-terminal kinase (JNK) are key markers of mitochondrial- and stimuli-mediated apoptosis.^36−39^ Remarkably, the cells incubated with the Dox-JNPs exhibited significant elevation of Caspase 3 and Caspase 9, while the other two groups showed comparably low levels. This finding was further corroborated by the expression of Apaf-1 and JNK, highlighting that the Dox-JNPs failed to alleviate the Dox-induced apoptosis of the cells. Conversely, the P-Dox-JNPs effectively mitigated the adverse effects of Dox, showing negligible change in the expression of these markers. To further substantiate our findings, we analyzed the survival and morphology of the cells via live/dead and actin/nucleus staining (Figures 4E and S15B). The cells incubated with the Dox-JNPs were mostly dead, with a significantly reduced spreading area of ca. 499.4 μm^2^ compared to that (ca. 9667 μm^2^) of the control group. In the case of the P-Dox-JNPs, the cells remained alive, displaying a spreading area of ca. 9318 μm^2^ comparable to that of the control. Taken together, the PEGylated, Dox-loaded Janus nanoparticles can serve as “cellular backpacks” with essentially no Dox-induced toxicity toward the carrier cells. Without PEGylation, however, the particles readily underwent endocytosis, leading to intracellular Dox release and subsequent cytotoxicity. With PEGylation, in contrast, the same batch of particles remains at the outer surface of the plasma membrane, spatially confining Dox to the extracellular space and thus minimizing its adverse impacts.

### Magnetic Manipulation and Evaluation of the Potential for Cell-Based Therapies

**Figure 5:**
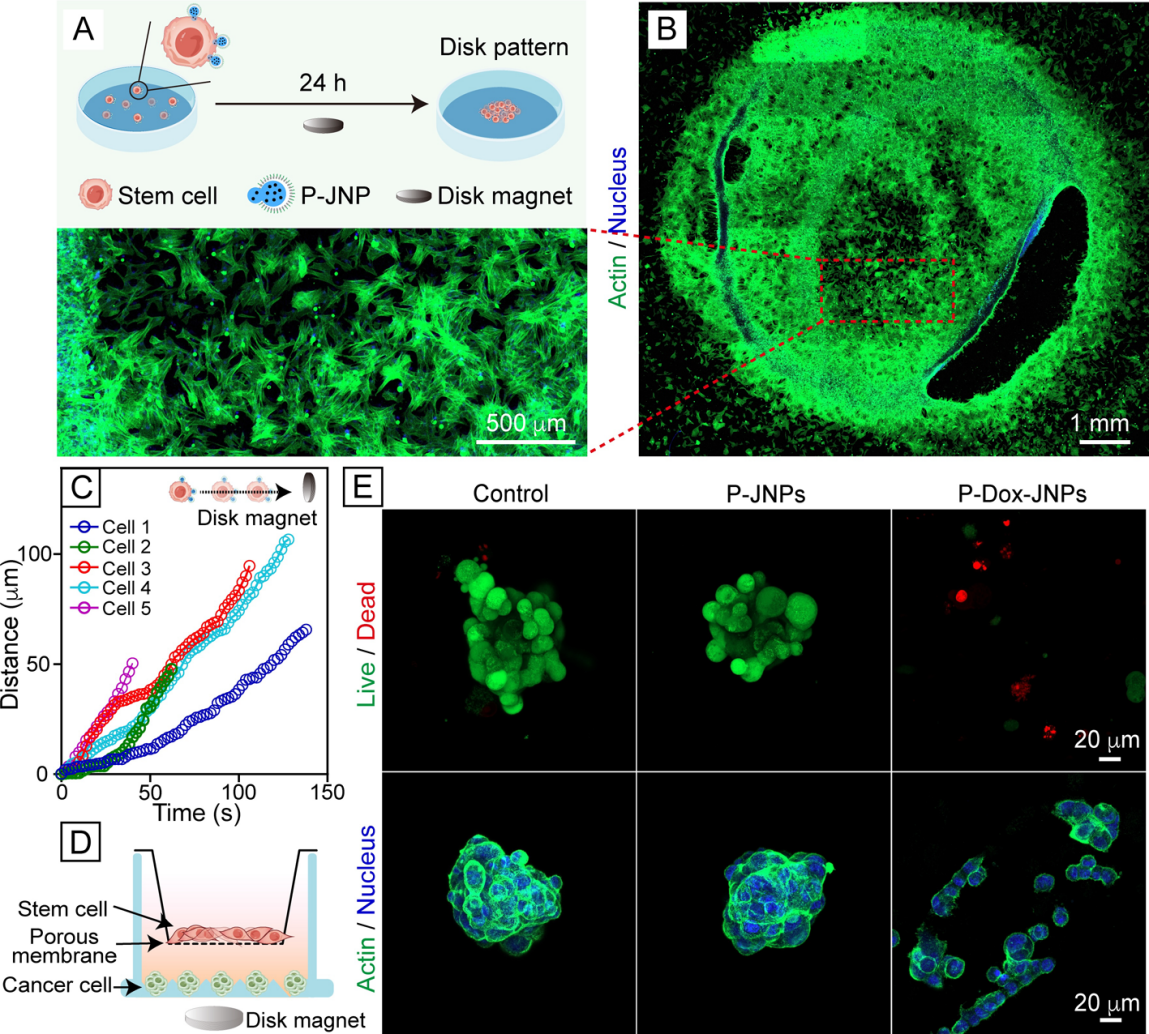
Magnetic manipulation and the therapeutic potential of MSCs carrying the drug-loaded Janus particles. (A,B) Schematic and confocal micrographs of MSCs decorated with the P-JNPs after subjection to a disk magnet placed beneath the culture dish, followed by actin/nucleus staining. (C) Magnet-directed migration of the MSCs decorated with the P-Dox-JNP particles. A disk magnet was placed next to a culture dish containing these cells to guide their migration. The inset illustrates the magnetic force exerted on these cells. (D) Schematic of the three-dimensional coculture system used for in vivo simulation. (E) Confocal micrographs of the Hela cancer cells in the lower chamber after 24 h of incubation with the MSCs (control) or P-Dox-JNP-decorated MSCs, followed by live/dead staining or actin/nucleus staining.

Given the strong magnetic responsiveness of the multifunctional Janus nanoparticles, we also investigated the behaviors of the MSCs decorated with the PEGylated particles under an external magnetic field. As illustrated in Figure 5A, the cells decorated with the P-JNPs were positioned above a disk magnet for 24 h and subsequently stained for actin and nucleus. It is interesting to note that the cells assembled into a disk pattern consistent with the magnetic field created by the disk magnet (Figure 5B). Prior studies have shown that a ring magnet can induce the alignment of magnetized cardiomyocytes along the field direction, leading to the formation of a ring pattern.^40^ Here we also cultured the cells decorated with the P-JNPs above a ring magnet for 2 h. The cells assembled into a ring and proliferated along this pattern (Figure S16). We then moved the magnet laterally by a small distance, followed by an additional 22 h of culture. In response to the magnetic field, some of the cells migrated and reorganized into a new ring, resulting in formation of a double-ring structure. These results demonstrate that the P-JNP-decorated cells can be manipulated by controlling the external magnetic field. To analyze the migration behavior of the P-JNP-decorated cells, we placed a disk magnet next to the culture dish and recorded the distributions of the cells as a function of time (Figure 5C). The cells progressively migrated closer to the magnet, with an average migration speed of ca. 1.04 μm/s for five cells randomly selected from the sample. This quick magnetic response allows for the secure anchor of particles to the plasma membrane of carrier cells before entering the tumor microenvironment. These results demonstrate that the carrier cells can migrate rapidly along the direction of an external magnetic field once they are decorated with the P-JNP.

It is established that cancer cells have higher metabolic activity than healthy cells for the production of excessive CO_2_ and lactate.^41^ This metabolic shift results in an acidified extracellular microenvironment to induce changes in the carrier particles through chemical bond hydrolysis or protonation of functional groups, thereby facilitating the release of preloaded drugs.^42^ In addition, the overexpression of tumor-associated enzymes, such as matrix metalloproteinases, cathepsins, phospholipases, and oxidoreductases,^43^ provides another avenue for drug release through enzyme-mediated reactions. These dynamic and heterogeneous factors collectively control the drug release kinetics in vivo. Herein, a Transwell coculture model involving cancer cells was utilized to evaluate the therapeutic potential of the P-Dox-JNP-decorated stem cells (Figure 5D). In this two-compartment model, cancer cells formed three-dimensional spheroids in the lower chamber to simulate the in vivo situation, whereas stem cells were seeded on the upper side of the porous membrane. Driven by the magnetic field, the stem cells carrying the drug-loaded P-Dox-JNPs quickly migrated toward the cancer cell spheroids (Figure S17). Since the SiO_2_ shell had a thickness of 40 nm, it could prevent premature and burst release of the loaded drug.^44^ The coating could stay intact until the carrier cells reached the acidic microenvironment of cancer cells to trigger the erosion of the SiO_2_ shell and thereby initiate Dox release.^41,45^ As shown in Figure 5E, the cancer cells within the spheroids underwent significant death and dissociation into the single-cell state, indicating the effective action of the Dox delivered using the “cellular backpacks”. Collectively, the multifunctional Janus nanoparticles not only enable a precise control over the migration of carrier cells, but also serve as “cellular backpacks” to facilitate targeted delivery.

In the present work, we focused on the fabrication and testing of cellular backpacks with an overall dimension of about 400 nm because larger particles (typically, several hundred nanometers in diameter) are known to be more effective in evading cellular internalization.^46^ The larger dimension also means that more therapeutic agents can be loaded and delivered per particle. Moving forward, it is important to optimize the key parameters, including the thickness of the SiO_2_ shell, the size of the mesopores in the SiO_2_ shell, and the dimensions of the two opposing halves of the Janus particles, to achieve the best performance in a specific application. Beyond the optimization in fabrication, future studies should be directed to the in vivo assessment of these Janus particles. This may include investigation of their interactions with the host immune system, circulation stability, biodistribution, and therapeutic efficacy. In particular, tracking these particles in vivo using MRI or fluorescence imaging will be essential for determining their retention and targeting efficiency. It is also crucial to investigate whether these particles remain firmly attached to the cell surface during circulation, as this could significantly impact their therapeutic efficacy and adverse impacts. The successful translation of these particles will depend on a comprehensive understanding of the biochemical properties of the drugs, the physiological functions of the carrier cells, and the pathological features of the diseases.

## Conclusions

We have demonstrated the fabrication of multifunctional Janus nanoparticles that can potentially serve as “cellular backpacks” for cell-based theranostics. Owing to their orthogonal surface properties, the Janus nanoparticles can anchor to the cell membrane by inserting the hydrophobic PS half into the lipid bilayer, while the PEGylated, hydrophilic SiO_2_ half points away from the cell surface. By design, the therapeutic agents such as Dox can be preloaded into the cavity of the SiO_2_ compartment and kept out of the plasma membrane to minimize their adverse impacts on the carrier cells. Additionally, the PS compartment can be loaded with SPIO nanoparticles to enable magnetic manipulation of cellular behaviors while enhancing T_2_-weighted MRI for image-guided treatment. Leveraging the multifunctionality of the Janus nanoparticles, we have demonstrated the targeted delivery of Dox with carrier stem cells to eradicate cancer cells in an in vitro spheroid model. In principle, the multifunctionality of the Janus nanoparticles also makes them a natural vehicle for developing combination therapies, such as those involving a combination of chemo (by Dox) and hyperthermia (by SPIO) treatments. Furthermore, the intrinsic properties of the carrier cells, including stem cells and immune cells, can also be separately engineered and then integrated with the multifunctional Janus nanoparticles to achieve the optimal therapeutic outcome. Altogether, this work offers a platform for the design and rational synthesis of multifunctional nanomaterials to advance cell-based theranostics.

## Supporting Information Available

Experimental details, characterizations of the nanoparticles, cytotoxicity measurements, and additional optical, confocal, and electron microscopy micrographs (PDF)
