# Supplementary material for: Multifunctional Janus Nanoparticles Capable of Anchoring to the Cell Membrane and Serving as “Cellular Backpacks” for Advanced Theranostics
Source: J Am Chem Soc. 2025 Apr 5;147(15):12973–81. doi: 10.1021/jacs.5c02587 (PMC12006992; doi:10.1021/jacs.5c02587)
Supplement: Supplementary file 1 — ja5c02587_si_001.pdf [file ja5c02587_si_001.pdf]

Supporting Information

Multifunctional Janus Nanoparticles Capable of Anchoring to the Cell Membrane and Serving as "Cellular Backpacks" for Advanced Theranostics

Min Hao,[†] Yidan Chen,[§] Johannes Leisen,[‡] Ted J. Whitworth,[∥] and Younan Xia[†, ‡, *]

[†]The Wallace H. Coulter Department of Biomedical Engineering, Georgia Institute of Technology and Emory University, Atlanta, Georgia 30332, United States

[§]School of Materials Science and Engineering, Georgia Institute of Technology, Atlanta, Georgia 30332, United States

[‡]School of Chemistry and Biochemistry, Georgia Institute of Technology, Atlanta, Georgia 30332, United States

[∥]Robert P. Apkarian Integrated Electron Microscopy Core, Emory University, Atlanta, Georgia 30322, United States

[*]Corresponding author. E-mail: younan.xia@bme.gatech.edu

EXPERIMENTAL SECTION

Chemicals and Reagents. Tetraethoxysilane (TEOS, ≥99.0%), iron (III) chloride hexahydrate ($FeCl_3 \cdot 6H_2O$, ≥98.0%), iron (II) chloride tetrahydrate ($FeCl_2 \cdot 4H_2O$, 98.0%), sodium dodecyl sulfate (SDS, ≥99.0%), rhodamine B (≥95.0%), potassium persulfate (KPS, ≥99.0%), oleic acid (90%), ammonium hydroxide ($NH_3 \cdot H_2O$, 28 wt%), tetrahydrofuran (THF, ≥99.9%), ammonium nitrate (NH_4NO_3, ≥99.0%), hexadecane (99%), octane (≥99.0%), 3-aminopropyl trimethoxysilane (APTMS, ≥98.0%), hexadecyltrimethylammonium bromide (CTAB, ≥99.0%), styrene (99.9%), N-hydroxysuccinimide (NHS, 98%), methoxy-polyethylene glycol-carboxylic acid (mPEG-COOH), and crystal violet solution (1%,v/v) were all obtained from Sigma-Aldrich. 1-ethyl-3-(3-dimethylaminopropyl) carbodiimide hydrochloride (EDC), paraformaldehyde, calcein AM, ethidium homodimer-1 (EthD-1), phosphate-buffered saline (PBS), Lyso Tracker™ Green DND-26, 4',6-diamidino-2-phenylindole (DAPI), Hoechst 33342, and penicillin-streptomycin were purchased from Thermo Fisher Scientific. Cell counting kit 8 (CCK8) and doxorubicin hydrochloride (Dox, >99%) were ordered from Dojindo and Biotang, respectively. Dulbecco's modified Eagle medium (DMEM) was purchased from the American Type Culture Collection (ATCC). Alpha minimum essential medium (α-MEM), High glucose DMEM (4.5 g/L D-glucose), and fetal bovine serum (FBS) were obtained from Gibco. TRIzol reagent was purchased from Invitrogen, and the QuantiTect reverse transcription kit was obtained from QIAGEN. CoraLite® Plus 488-Phalloidin (1:200, PF00001) was obtained from Proteintech. Ethanol (anhydrous, 99.5%) was ordered from VWR. Deionized (DI) water with a resistivity of 18.2 MΩ·cm at room temperature was used throughout the experiments.

Preparation of Ferrofluid in Octane. The ferrofluid was synthesized by following a reported protocol.[1,2] Briefly, 2.4 g of $FeCl_3 \cdot 6H_2O$ and 0.982 g of $FeCl_2 \cdot 4H_2O$ were dissolved in 10 mL of water under a nitrogen atmosphere with constant mechanical stirring at 300 rpm and 80 °C. Upon one-shot injection of 5 mL of $NH_3 \cdot H_2O$, the solution mixture turned black, indicating the formation of superparamagnetic iron oxide (SPIO) nanoparticles. The mixture was stirred for an additional 30 min before adding 0.376 g of oleic acid. The reaction was maintained at 80 °C for 1.5 h to enable surface modification. The resultant SPIO nanoparticles were washed sequentially with water (until pH=7) and ethanol to remove excessive oleic acid, and then dispersed in octane to obtain the ferrofluid with 65 wt.% concentration.

Synthesis of PS Beads Containing SPIO Nanoparticles (SPIO-PS). Mini-emulsions of ferrofluid and styrene were prepared separately.[1] To prepare the ferrofluid mini-emulsion, 0.004 g of SDS was dissolved in 4.8 g of water, followed by the addition of 0.2 g of the 65% ferrofluid. The mixture was sonicated using a Q125 ultrasonic processor (125 W, 20 KHz) for 15 min. For the styrene mini-emulsion, 0.005 g of SDS was dissolved in 4 g of water, followed by the addition of 0.25 g of styrene and 0.004 g of hexadecane, and the mixture also sonicated for 15 min.

Next, the ferrofluid mini-emulsion, styrene mini-emulsion, and 20 mg of KPS were combined in a three-neck flask and mechanically stirred at 200 rpm for 45 min under a nitrogen atmosphere. The reaction was performed at 80 °C for 20 h to obtain SPIO-PS. After the reaction, the nanoparticles were separated from the suspension by applying a magnet for 30 min to attract them to one side of the flask. After removing the supernatant, the products were washed three times with water by centrifugation at 5000 rpm for 5 min.

Preparation of the Multifunctional Janus Nanoparticles. 1 mL of the SPIO-PS beads (0.25%, w/v) was dispersed in a solution containing 0.018 g of CTAB, 4.025 mL of ethanol, 10.975 mL of water, and 0.1 mL of $NH_3 \cdot H_2O$. The mixture was stirred at 30 °C for 30 min, followed by the slow addition of 20 μL of TEOS. The reaction proceeded at 30 °C for 2 h, and the resulting particles were washed three times with ethanol *via* centrifugation (5000 rpm, 5 min). To functionalize the particle surface with amine groups, the particles were dispersed in 10 mL of ethanol containing 2% (v/v) APTMS and refluxed at 60 °C for 12 h, followed by washing three times with ethanol. Afterward, the particles were dispersed in 10 mL of a 20 or 30% (v/v) aqueous THF containing 1% (w/v) SDS and shaken at 300 rpm for 6 h for polymer swelling.[3] To quench the swelling of PS, 20 mL of ethanol was added, and the Janus nanoparticles were collected by centrifugation at 6000 rpm for 5 min and washed three times with ethanol. Then, these particles were dispersed in 10 mL of NH_4NO_3 in ethanol (10 mg mL^{-1}) and stirred at 60 °C for 2 h to remove CTAB.

To load a drug, the particles were dispersed in 10 mL of ethanol containing 1 mg mL^{-1} of Dox or rhodamine B and stirred overnight. The excess drug was removed by washing the sample with ethanol. For selective PEGylation,[4] the resulting particles were dispersed in 10 mL of ethanol with 45 mg of EDC and 30 mg of NHS, followed by the addition of 700 mg of mPEG-COOH. In this case, the amine groups on the SiO_2 half coupled with the -COOH groups of mPEG-COOH. The

mixture was stirred for 48 h, and the PEGylated particles were collected by washing sequentially with water and ethanol.

Cell Culture. Hela cells were obtained from ATCC and cultured in high glucose DMEM supplemented with 10% FBS and 1% penicillin-streptomycin. Rat tail tendon fibroblasts (provided by collaborators at Columbia University) were cultured in DMEM containing 10% FBS and 1% penicillin-streptomycin. Mesenchymal stem cells (MSCs) were purchased from a commercial source (Lonza, Basel, Switzerland) and maintained in α-MEM supplemented with 10% FBS and 1% penicillin-streptomycin. RAW 264.7 cells were ordered from ATCC and cultured in high glucose DMEM supplemented with 10% FBS and 1% penicillin-streptomycin. All cells were cultured at 37 °C in a humidified atmosphere with 5% CO_2. To ensure cell viability and experimental consistency, the cells were used only after being thawed and cultured for three passages.

Cell Live/Dead Staining. Cells were seeded at a density of 10^4 cells mL^{-1} in a 24-well culture plate. When the cells reached a confluence of *ca.* 70%, the culture medium was replaced with a fresh medium containing 40 μg mL^{-1} of the particles. After 24 h of incubation, the cells were treated with serum-free medium containing 2 μM calcein AM and 1 μM EthD-1 and then incubated at 37 °C for 20 min according to the manufacturer's instructions. Afterward, the samples were washed with PBS three times and observed using a confocal laser scanning microscope (CLSM). For the three-dimensional cell spheroids, the incubation time was extended to 1 h to ensure adequate penetration and staining of calcein AM and EthD-1 within the spheroids.

CCK8 Assay. Cells were seeded at a density of 10^4 cells mL^{-1} in the 96-well culture plate. After the cells reached a confluence of *ca.* 70%, the medium was replaced with the fresh medium containing the particles at varying concentrations. After 12 or 24 h of incubation, the medium was replaced with serum-free medium containing 10% CCK8 solution. Then, the cells were incubated at 37 °C for 1 h, and the absorbance of the medium was measured at 450 nm using a multi-mode plate reader (BioTek Synergy H1).

Observation of the Cell-Particle Interaction. Hela cells, fibroblasts, and MSCs were seeded at a density of 10^4 cells mL^{-1} in a 24-well culture plate, respectively. After the cells reached a confluence of *ca.* 70%, the medium was replaced with a fresh medium containing 40 μg mL^{-1} of the rhodamine B-labeled particles. After 3 or 24 h of incubation, the cells were washed with PBS three times before staining the membrane, lysosome, and nucleus. In detail, the cells were

incubated with CellBrite® Steady 488 (1:1000 according to the manufacturer's instructions) for 15 min to label the cell membrane, followed by a 5-min incubation with Hoechst 33342 (1 μg mL^{-1}) to label the cell nucleus. For lysosomal staining, the cells were incubated with Lyso Tracker™ Green DND-26 (60 nM) for 15 min, followed by a 5-min incubation with Hoechst 33342. Afterward, the cells were washed three times with PBS and observed under CLSM. The distribution of the particles, either inside the cell or on the membrane, was analyzed using ZEN Blue software. The co-localization of the particles with lysosomes was evaluated using the plot profile function in Image J.

For transmission electron microscopy (TEM) observation, the samples were washed with PBS and fixed in a mixture of 2.5% glutaraldehyde, 1.0% paraformaldehyde, 0.03% picric acid, and 0.03% calcium chloride in 0.1M cacodylate buffer for 2 h at room temperature. The samples were then washed in 0.1M cacodylate buffer and refrigerated at 4 °C until further processing. Afterward, the samples were post-fixed in 1% osmium tetroxide/cacodylate buffer for 1 h and rinsed in cacodylate buffer (pH=7.4) three times. Subsequently, the samples were dehydrated through a graded ethanol series (50%, 70%, 95%, and 100%) at room temperature, infiltrated with epoxy resin overnight, and then polymerized at 60 °C for 48 h. Ultrathin sections (70 nm) were prepared using a Leica EM UC6 ultramicrotome. Finally, the sections were stained with aqueous uranyl acetate and lead citrate prior to imaging.

Reverse Transcription-Quantitative Polymerase Chain Reaction (RT-qPCR) Analysis. Cells were seeded at a density of 10^4 cells mL^{-1} in the 6-well culture plate. After the cells reached a confluence of *ca.* 70%, the medium was replaced with a fresh medium containing the particles at varying concentrations, as specified in the main text. After 24 h of incubation, the cells were lysed using TRIzol reagent to extract the total RNA. The total concentration and purity of extracted RNA were measured using a spectrophotometer (Nanodrop, Thermo Fisher Scientific). We then added cDNA, primers, and SYBR Green qPCR Master Mix into 96-well PCR plates. The thermal cycling was performed using StepOnePlus™ Real-Time PCR System (Applied Biosystems). The expression of relative genes was calculated using the ΔΔCt method. Primer sequences are provided in Supplementary Table 1.

Immunofluorescence Staining of Actin and Nucleus. Cells were seeded following the same procedure described for live/dead staining. After 24 h of incubation, the cells were washed with PBS and fixed with 4% paraformaldehyde for 15 min. Subsequently, the cells were permeabilized

using 0.2% Triton X-100 for 5 min and washed three times with PBS. Afterward, the samples were incubated with CoraLite® Plus 488-Phalloidin (1:200) for 20 min, followed by staining with DAPI (1 μg mL^{-1}) in PBS for 5 min. Finally, the samples were washed three times with PBS and observed under CLSM.

Magnetic Responsiveness of Cells. A cell suspension at a density of 10^4 cells mL^{-1} was incubated with 40 μg mL^{-1} of the PEGylated Janus nanoparticles for 3 h to ensure their anchoring to the cell membrane. These cells were subsequently cultured in a 29 mm diameter dish (D29-20-1.5H, Cellvis) with a disk magnet (Neodymium, LOVIMAG) positioned beneath the dish. After 24 h of incubation, the samples were rinsed three times with PBS and stained for actin and nucleus using CoraLite® Plus 488-Phalloidin and DAPI, respectively. Alternatively, to generate a double-ring pattern, a ring-shaped magnet was placed beneath the dish for 2 h and then laterally moved to another position and held there for 22 h. After rinsing three times with PBS, the samples were stained with CoraLite® Plus 488-Phalloidin and DAPI, respectively. To analyze cell migration, we placed the cells in a petri dish of 29 mm in diameter, with a magnet positioned at one side of the dish. Cell migration was recorded and tracked using optical microscopy, and the migration speed was analyzed using Image J software.

Construction of the Co-Culture Systems. We utilized a Transwell permeable support (pore size: 8 μm) to establish the co-culture system. A Transwell permeable support consists of a porous membrane and a fixed support structure. MSCs at a density of 10^4 cells mL^{-1} were seeded onto the porous membrane of Transwell permeable support. Simultaneously, Hela cells were seeded in AggreWell 24-well plates (Stemcell) according to the manufacturer's instructions to form cell spheroids. After overnight incubation, the PEGylated Janus particles were added into the Transwell permeable support and incubated for 3 h to ensure that the particles anchored to the cell membrane. Afterward, the Transwell permeable support was transferred onto the well containing the Hela cells, with a magnet placed underneath the well.

To visualize the cells that migrated from the upper side to the underside of the porous membrane, the cells remaining on the upper side were carefully removed using a cotton swab to prevent imaging interference. The cells on the underside were then fixed with 4% paraformaldehyde for 15 min and stained with 0.1% crystal violet solution for 15 min. After washing with PBS three times, the samples were imaged under an optical microscope.

Characterizations. TEM images were acquired using HT7700 TEM (Hitachi, Japan). Scanning electron microscopy (SEM) images were obtained using SU8230 SEM (Hitachi, Japan). Zeta potential measurements were performed using Zetasizer Nano ZS (Malvern Instruments, UK). Fluorescence micrographs were captured using CLSM 900 (Zeiss Co., Germany). Ultraviolet-visible (UV-vis) spectrum was recorded on a Cary 60 UV-vis spectrometer (Agilent Technologies, USA). The T_1 and T_2 relaxation times of the particles were measured using MARAN 23 (Resonance Instruments, Oxford, UK), while T_2-weighted MR images were obtained using Bruker Pharmascan MRI system (Bruker, Germany) operated at a magnetic field strength of 7 T with a solenoid coil of 38 mm in inner diameter. A multi-slice multi-(spin) echo (MSME) sequence protocol was employed with an echo time (TE) of 6.84 ms and a matrix of 128x128 pixels.

Statistical Analysis. Statistical analyses were performed using GraphPad Prism 10 and ImageJ. Significance differences were assessed using one-way ANOVA (for a single independent variable) or two-way ANOVA (for multiple independent variables), followed by Tukey's comparison test and defined as $p < 0.05$. Sample sizes (n) are specified in the corresponding figure captions.

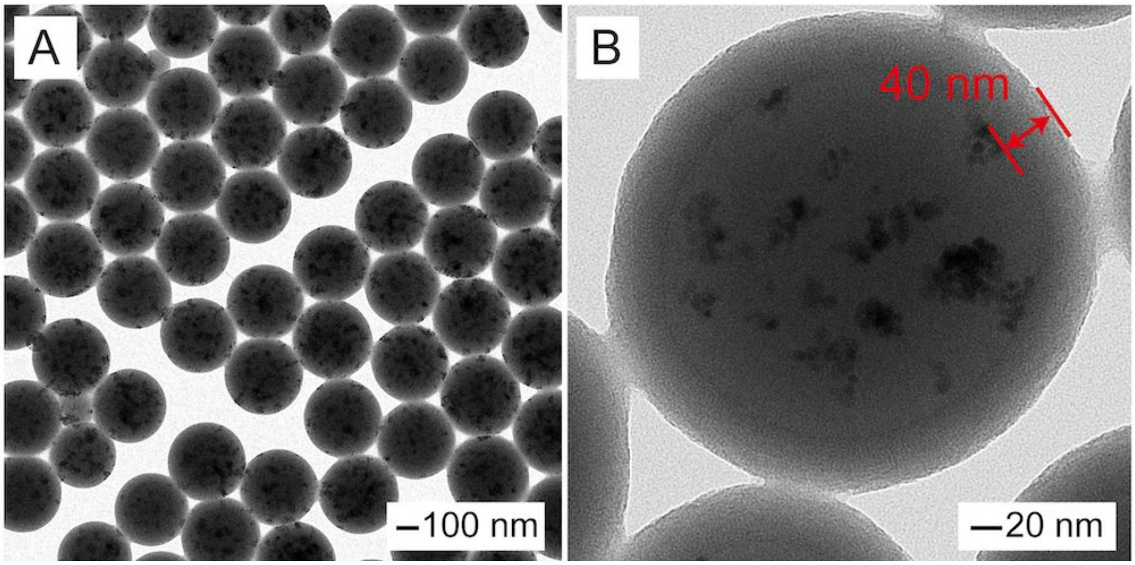

Figure S1. TEM images of the (A) SPIO-PS beads and (B) (SPIO-PS)@SiO_2 core-shell nanoparticles.

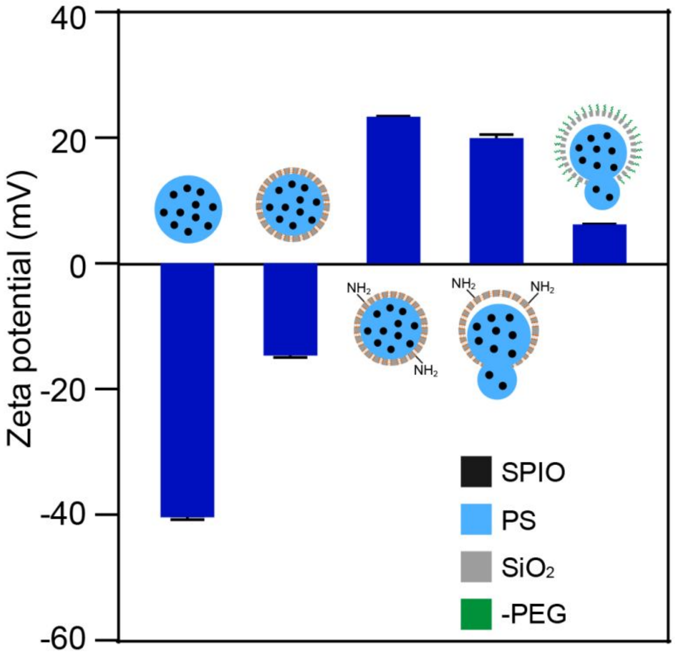

Figure S2. Zeta potentials of the samples at different stages of the multifunctional Janus nanoparticle preparation.

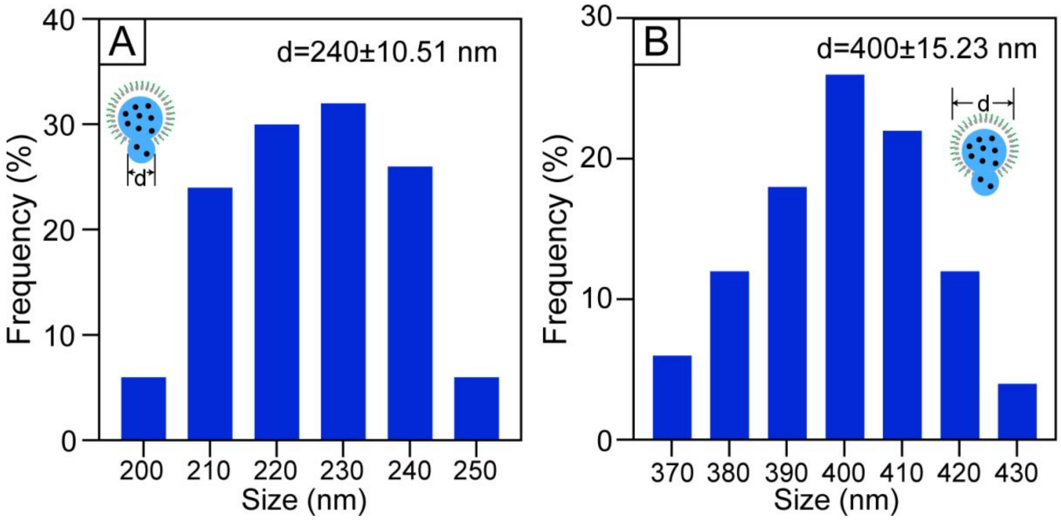

Figure S3. Size distributions of the (A) PS half and (B) SiO_2 half of the multifunctional Janus nanoparticles. The diameters were derived from SEM images of the particles (n=50).

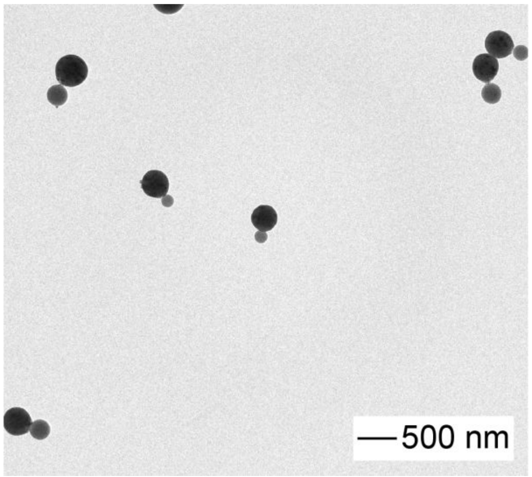

Figure S4. TEM image of the multifunctional Janus nanoparticles fabricated by swelling the PS core with aqueous THF (30%, v/v) for 6 h, followed by quenching the sample with ethanol, removal of CTAB, and PEGylation.

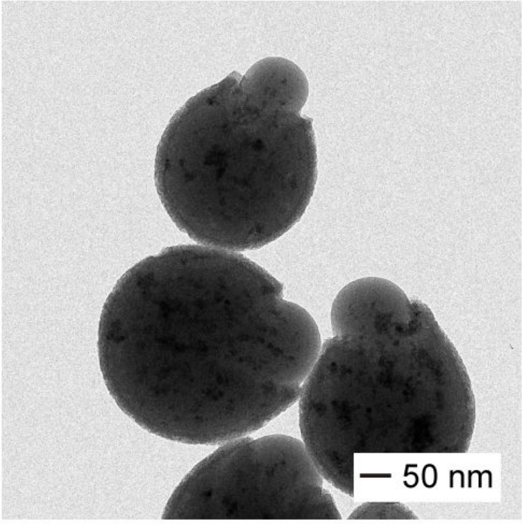

Figure S5. TEM image of the multifunctional Janus nanoparticles fabricated by swelling the PS core with aqueous THF (20%, v/v) for 6 h, followed by quenching the sample with ethanol, removal of CTAB, and PEGylation.

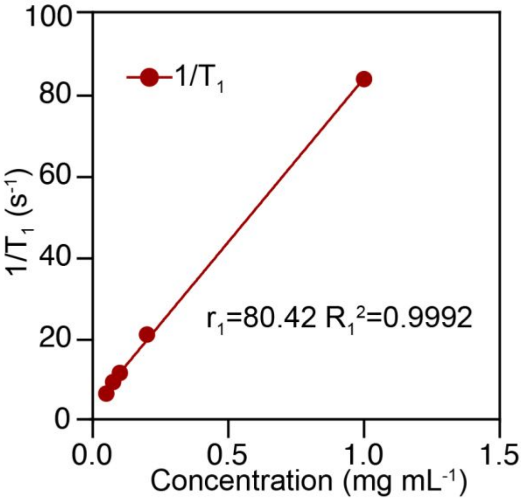

Figure S6. Plot of $1/T_1$ of the multifunctional Janus nanoparticles as a function of the particle concentration.

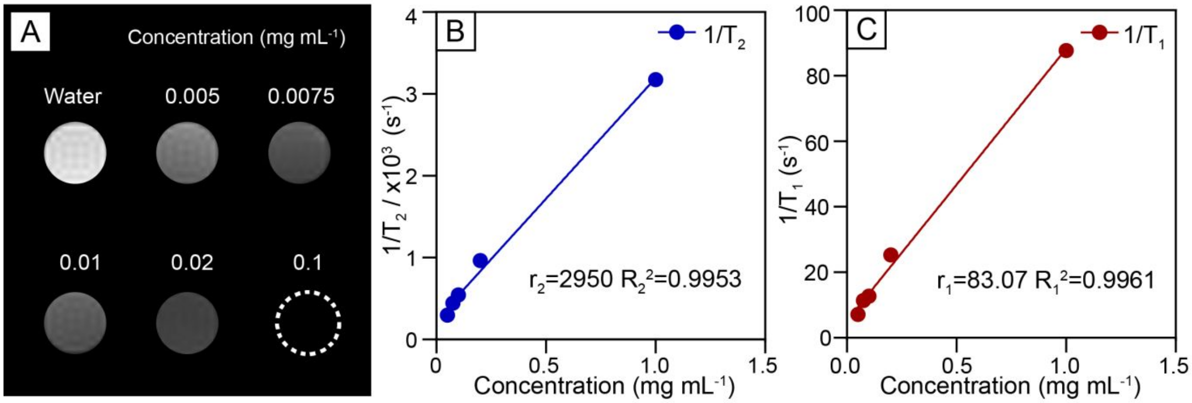

Figure S7. (A) T_2-weighted MRI, (B) plot of $1/T_2$, and (C) plot of $1/T_1$ of the (SPIO-PS)@SiO_2 core-shell nanoparticles as a function of particle concentration.

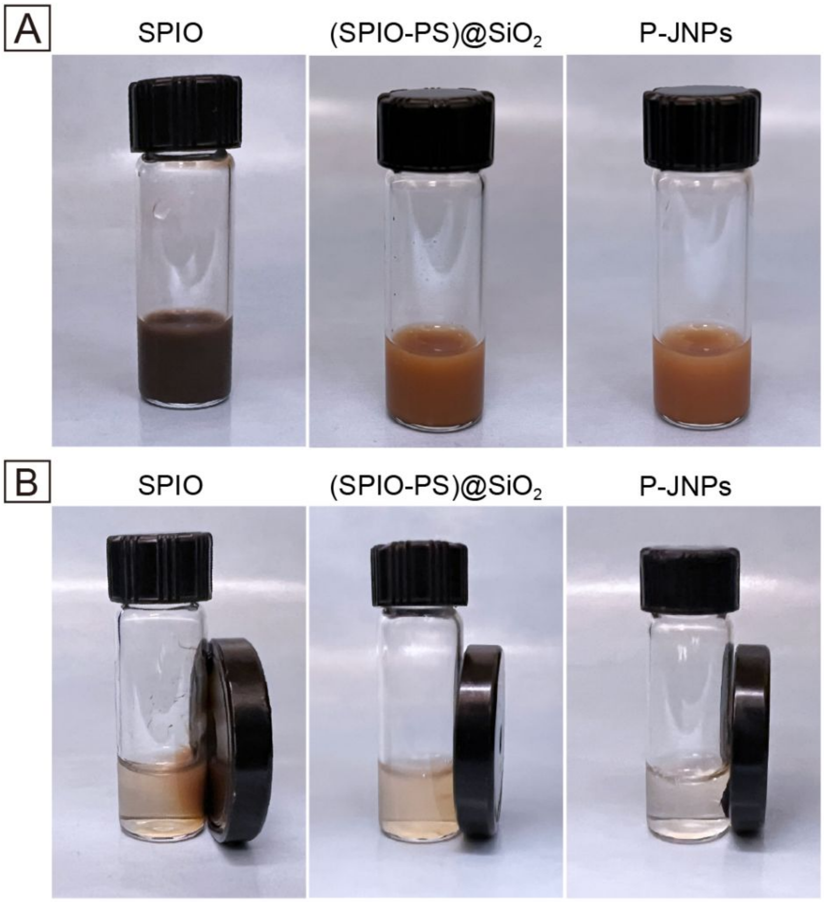

Figure S8. Digital photographs of the samples (A) before and (B) after applying a magnet to one side of the container.

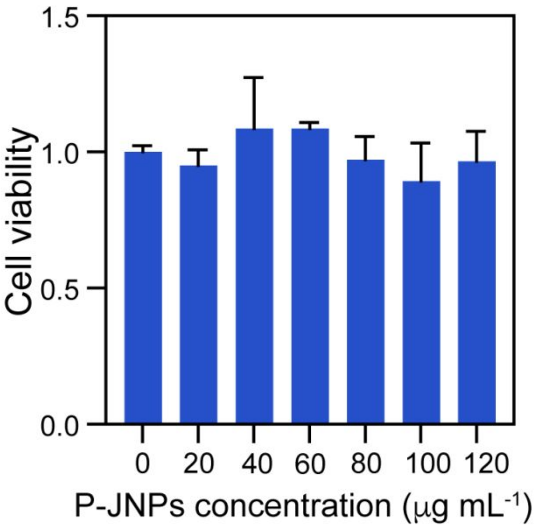

Figure S9. CCK8 assay evaluating the viability of MSCs after incubation with P-JNPs at varying concentrations for 24 h.

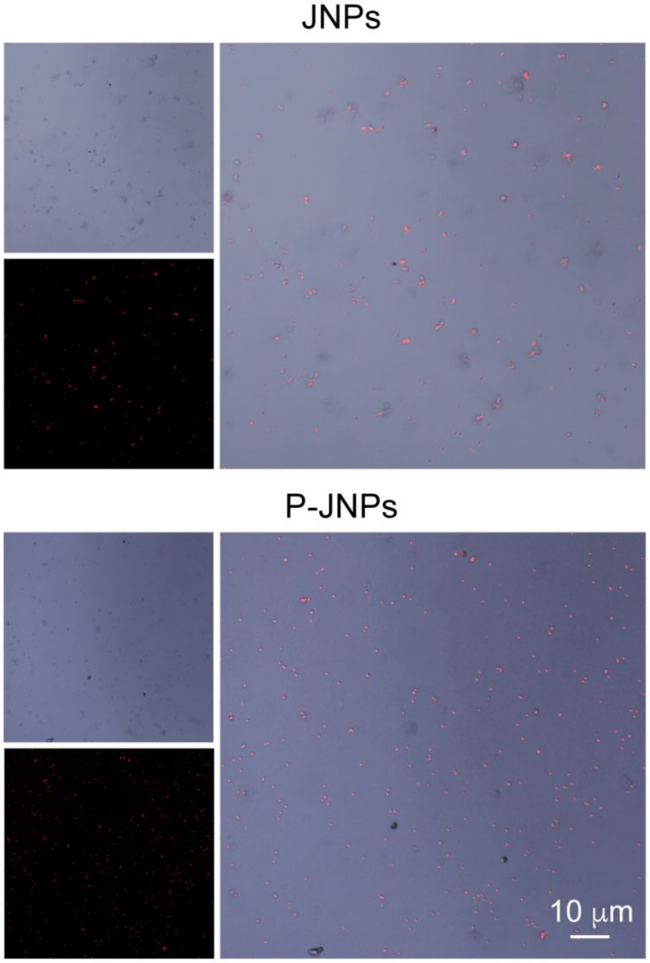

Figure S10. Bright-field (top left), fluorescence (bottom left), and merged (right) micrographs of the two types of rhodamine B-labeled Janus nanoparticles.

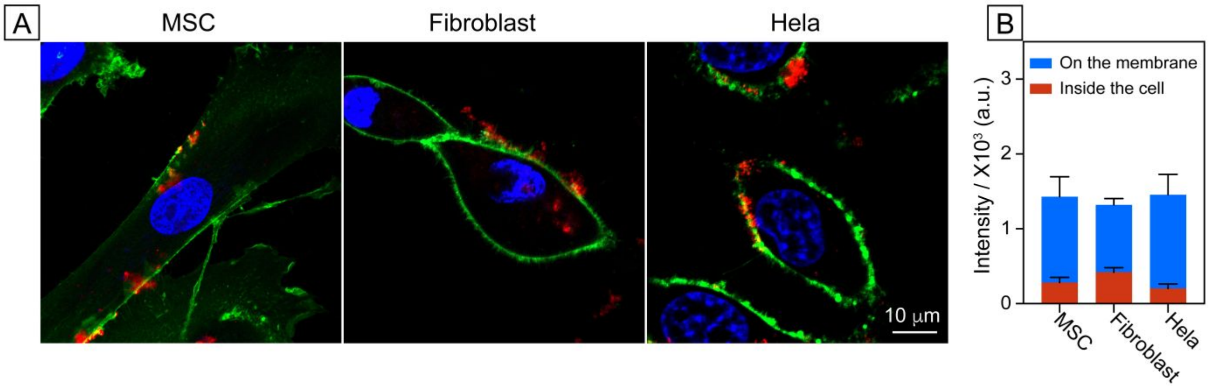

Figure S11. (A) Confocal fluorescence micrographs of the cells incubated with P-JNPs featuring a smaller (*ca*. 170 nm) PS protrusion relative to the particles shown in the main text for 24 h, followed by staining of the plasma membrane with CellBrite®steady (green) and nucleus with Hoechst 33342 (blue). The particles were labeled with rhodamine B for red fluorescence. (B) A quantitative analysis of the distributions of the P-JNPs with the smaller PS protrusion situated on the plasma membrane and inside the cell, respectively. Data are shown as mean ± SD (n = 10).

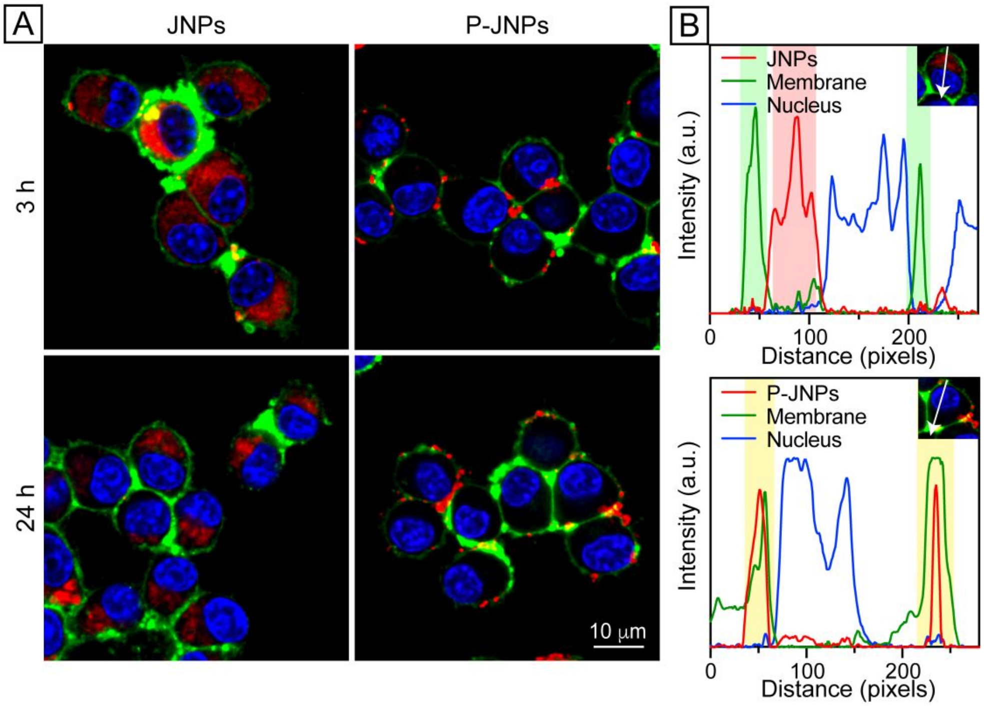

Figure S12. (A) Confocal fluorescence micrographs of the RAW 264.7 cells incubated with the two types of Janus nanoparticles (red) for 3 and 24 h, respectively, followed by staining of the plasma membrane with CellBrite®steady (green) and nucleus with Hoechst 33342 (blue). (B) Profiles of fluorescence intensities of the two types of particles, the plasma membrane, and nucleus in an individual cell after 24 h of incubation. The fluorescence profiles correspond to the white arrows in the insets. The red, green, and yellow-colored boxes represent the primary fluorescence distribution regions of the particles, plasma membrane, and their overlap, respectively.

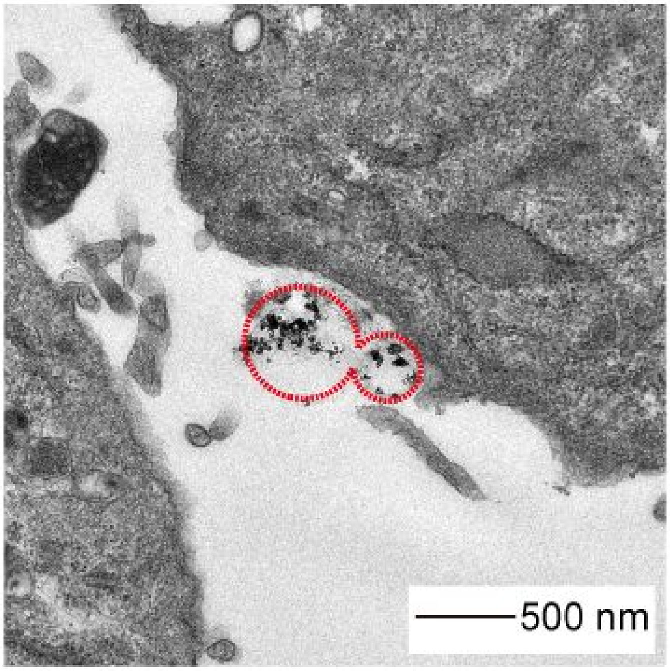

Figure S13. TEM image showing the MSC in contact with one of the P-JNPs (outlined by red contours).

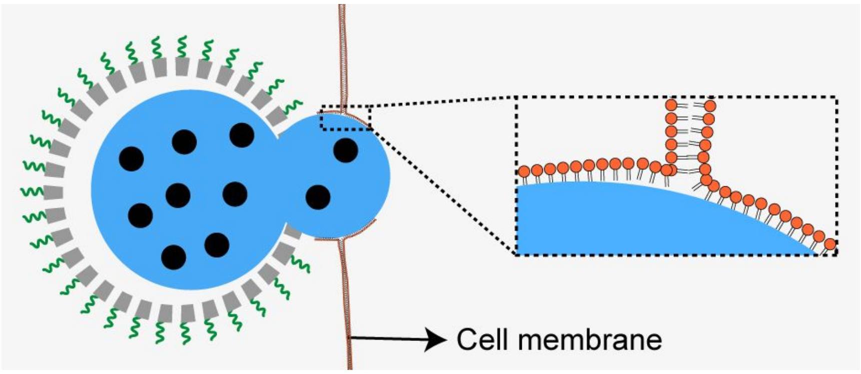

Figure S14. Schematic showing the interaction between the P-JNP and cell membrane. Note that the cell membrane is much thinner than the dimensions of the nanoparticle.

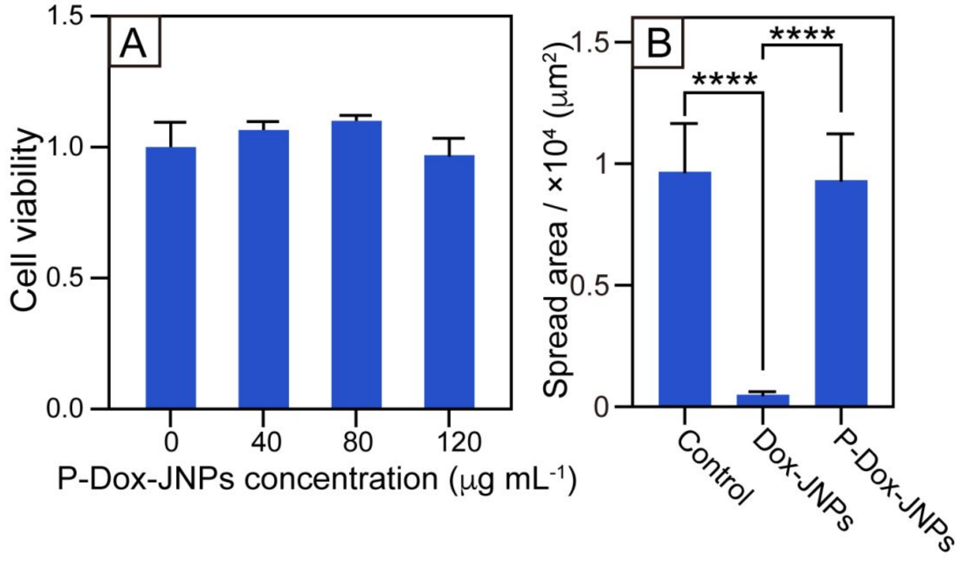

Figure S15. (A) CCK8 assay evaluating the viability of MSCs after incubation with P-Dox-JNPs at varying concentrations for 24 h. (B) A quantitative analysis of the cell spreading behaviors after incubation with the two types of Dox-loaded particles for 24 h.

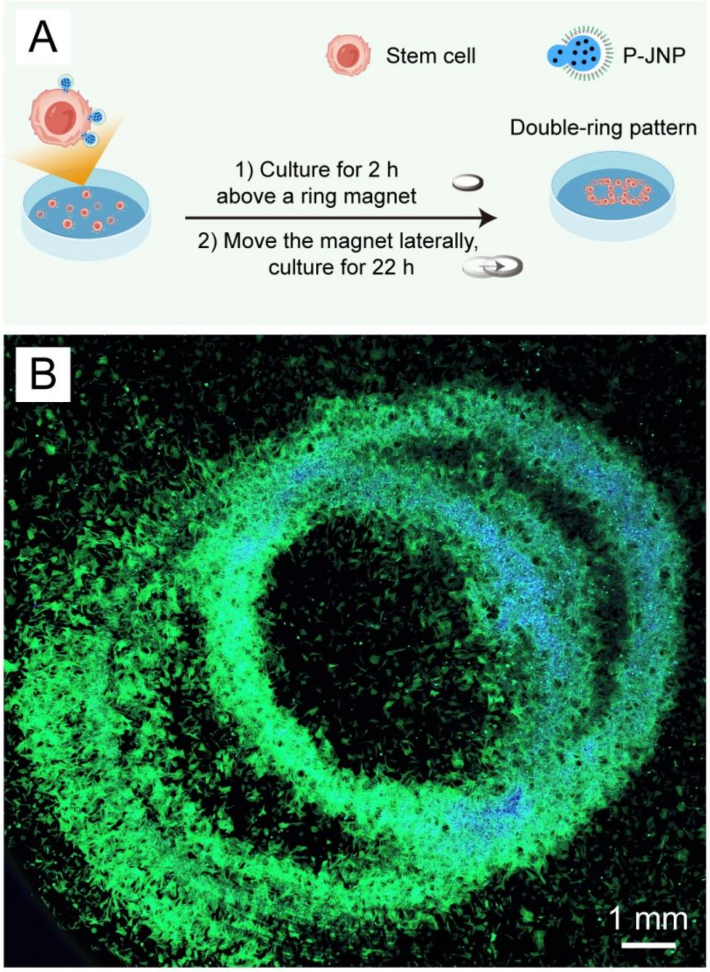

Figure S16. (A) Schematic and (B) confocal micrographs of the MSCs decorated with the P-JNPs after culture above a ring magnet for 2 h, followed by moving the magnet laterally and an additional 22 h of culture. Actin and nucleus staining were performed after culture.

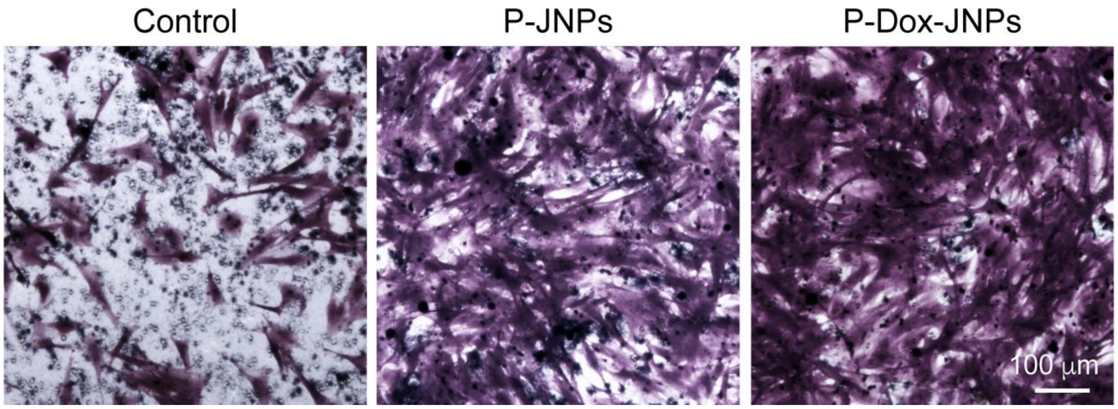

Figure S17. Optical micrographs of crystal violet-stained MSCs on the underside of the porous membrane in Transwell co-culture model following 3 h of magnetic guidance. Increased cell density in the P-JNPs and P-Dox-JNPs groups compared to the control indicated enhanced MSC migration under magnetic guidance.

Table S1. The sequences of primers for RT-qPCR.

Gene	Forward primers (5’-3’)	Reverse primers (5’-3’)
β-actin	CATGTACGTTGCTATCCAGGC	CTCCTTAATGTCACGCACGAT
Caspase 3	GAAATTGTGGAATTGATGCGTGA	CTACAACGATCCCCTCTGAAAAA
Caspase 9	CTCAGACCAGAGATTCGCAAAC	GCATTTCCCCTCAAACTCTCAA
Apaf-1	GTCACCATACATGGAATGGCA	CTGATCCAACCGTGTGCAAA
JNK	CTGCGTCACCCATACATC	TGGCGTTGCTACTTACTGC

REFERENCES

(1) Xu, H.; Cui, L.; Tong, N.; Gu, H. Development of High Magnetization Fe_3O_4 /Polystyrene/Silica Nanospheres via Combined Miniemulsion/Emulsion Polymerization. *J. Am. Chem. Soc.* **2006**, *128*, 15582–15583.

(2) Xu, Y.; Xu, H.; Gu, H. Controllable Preparation of Epoxy-functionalized Magnetic Polymer Latexes with Different Morphologies by Modified Miniemulsion Polymerization. *J. Polym. Sci., Part A: Polym. Chem.* **2010**, *48*, 2284–2293.

(3) Qiu, J.; Chen, Z.; Chi, M.; Xia, Y. Swelling-Induced Symmetry Breaking: A Versatile Approach to the Scalable Production of Colloidal Particles with a Janus Structure. *Angew. Chem., Int. Ed.* **2021**, *60*, 12980–12984.

(4) Li, X.; Li, Y.; Yu, C.; Bao, H.; Cheng, S.; Huang, J.; Zhang, Z. ROS-Responsive Janus Au/Mesoporous Silica Core/Shell Nanoparticles for Drug Delivery and Long-Term CT Imaging Tracking of MSCs in Pulmonary Fibrosis Treatment. *ACS Nano* **2023**, *17*, 6387–6399.
